# Gestalt Theory Rearranged: Back to Wertheimer

**DOI:** 10.3389/fpsyg.2017.01782

**Published:** 2017-10-11

**Authors:** Shelia Guberman

**Affiliations:** Retired, Cupertino, CA, United States

**Keywords:** Gestalt theory, basic laws, terms, ambiguity, imitation principle

## Abstract

Wertheimer's seminal paper of 1923 was of gerat influence in psychology and other sciences. Wertheimer also emphasized the weaknesses of the newborn Gestalt theory: too many basic laws, and the ambiguity of definitions. At the same time, the paper contained potential solutions to these problems, in the form of a number of very important ideas, some of which were presented implicitly: perception through imitation, communicative nature of linear drawings and writings, transfer from the visual domain to motor domain, linguistic interpretation of the Gestalt. In this paper it will be shown that based on these ideas the Gestalt theory can be rearranged so that the main notions can be well defined, and the general principle of Gestalt perception, which overarches all known laws and unifies different Gestalt phenomena **(the imitation principle)** can be introduced. The presented model of Gestalt perception is supported by fundamental neurophysiological data—the mirror neurons phenomenon and simulation theory.

## Introduction

Wertheimer's paper of 1923 formulated the fundamental problems and basic laws of visual perception. His ideas were so deep and constructive that for the following century they determined the course of Gestalt psychology and influenced other areas of psychology and other sciences. What is particularly striking about Wertheimer's paper is that the author himself pointed out the weaknesses of the new theory.

1. While presenting a number of very powerful laws of grouping, Wertheimer demonstrated that in many situations they contradict each other. This was the reason why he named them not laws but Factors.
2. Wertheimer understood that the bigger is the number of basic principles the weaker is the theory, but he didn't suggest a general principle that covers all other Factors.
3. Wertheimer understood and even emphasized the fuzziness of the definitions of the basic notions, and referred to reader's intuition by using such expressions as “one has a feeling how successive parts should follow one another”; “one knows what a ‘good’ continuation is, how ‘inner coherence’ is to be achieved, etc.”; “one recognizes a resultant ‘good Gestalt’ simply by its own ‘inner necessity’”.

A year later, Wertheimer emphasized the problem of ill-defined terms: “The attempt to explain Gestalt theory in a short essay is the more difficult because of the terms which are used: part, whole, intrinsic determination. All of them have in the past been the topic of endless discussions where each disputant has understood them differently” (Wertheimer, 1924).

Based on a number of ideas, which were neglected by his followers, it was possible:

redefine the basic notions of Gestalt psychology (such as whole, parts, Gestalt),

specify the domain of applicability of Gestalt theory of perception as communications,

introduce the general principle of perception – the imitation principle,

identify the mirror neurons phenomena (Ferrary et al., 2005; Iacoboni et al., 2005) as the neurologi-cal basis of imitation principle,

unite Gestalt phenomena from different domains (video, audio, music, speech, apparent.

movement) under one principle.

## Definitions of basic notions

The definitions of basic notions of Gestalt psychology have been a persistent target of criticism from many psychologists and non-psychologists including some of the greatest minds in physics and mathematics. Einstein pointed out the problem of poor definitions as a weakness of the whole of psychology. This is what he wrote in the obituary for Ernst Mach:

“Physics and psychology are to be distinguished from each other not by the objects they study but only by the manner of ordering and relating them. The activity of ordering yields abstract concepts and laws (rules). Concepts only have sense according to Mach to the extent that things can be shown to be interrelated, as well as **clearly** arranged. The people who have failed to analyze their own concepts will raise energetic protests and complain about the revolutionary threat to their holy property” (Einstein, 1916).

In 1946 the inaugural Macy Conference took place. It was entitled “Feedback Mechanisms and Circular Causal Systems in Biological and Social Systems,” and was the first ‘coming together’ of the hard scientists and the social scientists. The Core group included such luminaries in mathematics, computers, psychology and neurology as von Neumann, Wiener, McCulloch (chair), Pits, Lewin, Klüver, Northrop, Rosenblueth, Bateson, and Bigelow. Von Neumann and Wiener recommended that **the concepts of “field,” “Gestalt,” and others be clarified**. The main outcome of this discussion was an illustration of **how little the attendees agreed on the definitions and implications of these labels**.

The same fight for hard scientific principles could be observed at the philosophical level. In Davos (1929) at the famous “zurueck zu Kant” gathering Cassirer opposed Heidegger emphasizing that philosophy must retain the spirit of critical inquiry, the openness to natural science, and the clarity of rational argumentation that marked Kant himself as a great philosopher” (Gordon, 2010).

The demands of Einstein, von Neumann, Wiener, and Wertheimer himself were rejected. The issue has still not been resolved.

Here are the existing definitions of some basic notions of Gestalt theory.

***Whole and parts***. From very beginning and until now these notions stay undefined. “Part and whole have in the past been the topic of endless discussions where each disputant has understood them differently” (Wertheimer, 1924).

***Gestalt***. Today the popular definition of Gestalt is: “An organized whole that is perceived as more (or another) than the sum of its parts”. But what is the *sum*?

Other definitions are:

- Something that is made of many parts and yet is somehow more than or different from the combination of its parts.
- When you put the parts together, you get the whole - in other words, the *Gestalt*.
- *Gestalt* is a psychology term, which means “unified whole”.
- *Gestalt* means ‘seeing the whole picture all at once’.
- Ehrenfels: By means of *Gestalt* qualities, we mean such positive connotations, which are connected with the presence of conceptual complexes in the consciousness, which, in turn, consist of elements which can be separated from each other (i.e, imaginable without one another).
- Köhler in his book *Gestalt Psychology* (1947) presented two controversial definitions: “it has the meaning of a concrete entity *per se*”, and “the segregation of specific entities in the sensory field”. In the first definition, the Gestalt is an object from the outside world; in the second one it belongs to the psychological domain. Such conflations can be found in many Gestalt psychology papers and have destroyed many theoretical constructions^1^

All these statements are not operational.

***Good:*** “The term ‘good’ is undefined but is generally regarded as embracing such properties as regularity, symmetry, simplicity” (Koffka, 1935).

***Good Gestalt****:* simple, orderly, balanced, unified, coherent, regular, etc. *ad infinitum*.

***Bad Gestalt*** is “defined” through good Gestalt, which is itself ill defined.

***Isomorphism*** in Gestalt literature has many (sometimes controversial) meanings (see critical review by Luchins and Luchins, 1999).

**Figure 1:**
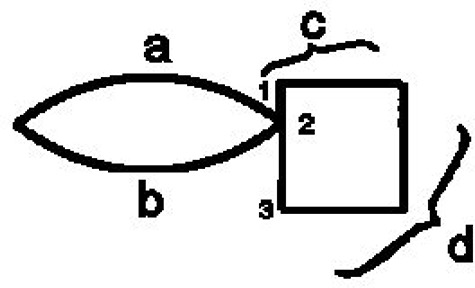
Law of closure (Wertheimer, 1923).

***Closure***. Wertheimer introduced the Factor of Closure to resolve the perception of the only example (Figure 1).

**Figure 2:**
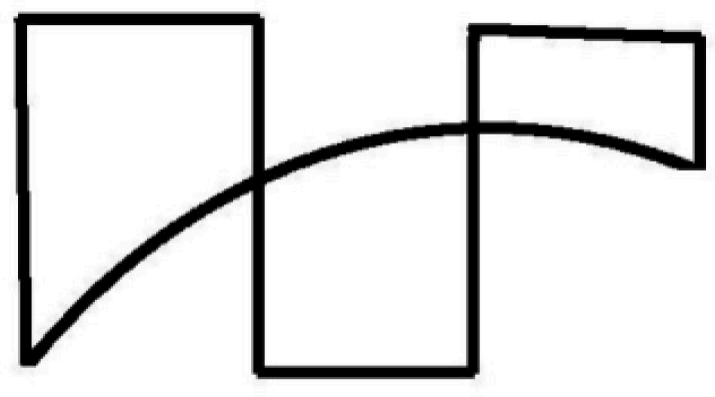
Contradiction between laws of closure and good continuation (Wertheimer, 1923).

Right after this he shows that this Factor is beaten by the good continuation Factor (Figure 2). Different writers used this term with different meanings. Luchins and Luchins in their critical review came to a remarkable conclusion: “We advocate a **moratorium** on the use of this term closure despite the current popularity of Gestalt terminology” (Luchins and Luchins, 1959). The response of the Gestalt community to the criticism was even more remarkable—complete silence for 60 years.

## Multiplication of grouping laws

Despite Wertheimer's intention to avoid building Gestalt theory on multiple particular and mutually contradictory laws, many new grouping laws were introduced:

good form,
extension of common fate,
synchrony,
common region,
connectedness,
uniform connectedness,
space-time coupling,
contour grouping,
main orientation,
directional symmetry,
convexity,
past experience,
and more.

Every time gestaltists found an example that could not be explained by one of the existing Gestalt laws, a new law was introduced (the last of these new laws was suggested in 2010 Pinna, 2010).

## The general principle of prägnanz

For many years the principle of Prägnanz has been proclaimed as the main principle overarching all other grouping principles, but, as we showed in the previous paragraph, it never works, and new partial laws have continued to be suggested. This is because the Prägnanz law was never defined. It has many contradictory translations and interpretations, but not a single clear definition:

- tendency to the good Gestalt (the most popular description despite the fact that the terms *good* and *Gestalt* are not defined),
- the Prägnanz tendency is a general striving toward order and unity (Metzger),
- “Psychological organization will always be as ‘good’ as the prevailing conditions allow” (Koffka),
- tendency to a prägnant configuration (Luccio),
- mysterious tendency toward pragnanz (Verstegen),
- tendency towards Pragnanz der Gestaltung (Luchins&Luchins),
- laws of pragnanz are laws of clarity (Dewey),
- tendency towards simple arrangement (Wagemans),
- simplicity or Prägnanz (Fuchs),
- tendency to capture the essence of what we perceive (Revlin),
- Prägnanz or simplicity principle (van der Helm).

According to U. Neisser, “Köhler was well aware of and embarrassed by the circularity of the ‘law of Pragnanz’. He never stopped hoping that a better definition would be found. When I took ‘the Köhler seminar’ at Swarthmore in 1952, decades after *Die Physischen Gestalten*, one of the first tasks he put before us was to suggest definitions for ‘Pragnanz.’ I don't recall that we had anything useful to say”(Neisser, 2002).

The statement “Prägnanz is a tendency to the good Gestalt” was attributed to Wertheimer's classic paper (1923) (Wagemans et al., 2012), but in this paper Wertheimer used the word *Prägnanz* as an attribute of the word *Stuffen* only, and the expression “tendency to a good Gestalt” was used not as a definition, but as a reference to our intuition. *Prägnanz* has had many arbitrary translations. The straight translation of *Prägnanz* is “short description”, and this is what Wertheimer had in mind, though nobody paid attention.

In the big review of the history of Gestalt theory Wagemans et al. (2012), the above-mentioned weaknesses of Gestalt theory are admitted. One of the cardinal problems investigated by the authors (the group of leading scientists in Gestalt psychology) is the substitution of many local grouping laws by a single one encompassing all the others. For a long time it was the law of Prägnanz, but the review qualified it as intuitive, and therefore not operational: it was impossible to apply this law to any particular stimulus. Therefore the authors were forced in the first part of the review to present the analysis of dozens of old and new grouping laws. The attempts to modernize the general principle of Prägnanz are presented in the second part of the review: simplicity principle, minimum principle, and likelihood principle. The authors noted that despite the use in these new notions of mathematical formulas, the variables (simplicity, probability, likelihood) are ill defined, and can't be measured. The authors also remark that the notion of Prägnanz can be further substantiated in terms of the intrinsic dynamics of the brain as a self-organizing, adaptive system.

It has to be mentioned that the notion of Prägnanz and all subsequent models were proposed under the common assumption that there exists a space of bad Gestalten, in which one point (the good Gestalt) has to be found. Unfortunately, this space was never defined, and never presented. This is why the reviewers were not satisfied with all the mentioned hypotheses, and at the end of the review gave up: “Concepts developed closely to experience are not easily expressed in any formalism”. In the following paragraphs I will present a model, which shows that when perceiving the communicative stimuli we get only one percept—the good Gestalt.

## Back to wertheimer

Wertheimer's paper contains a number of very important ideas, which were presented implicitly, and had the potential to resolve the weaknesses of Gestalt theory:

1. The idea of particularity of perceiving the communicative visual signals (dotted and linear drawings), which implies the existence of another human being (the sender of the message).
2. The idea of “perceiving through imitation” – the future *simulation theory of perception* based on the mirror neurons phenomenon, which revolutionized the theory of perception.
3. To characterize the *good Gestalt* Wertheimer used the term *Prägnanz*, which means “short description”.

A number of fundamental problems in other soft sciences (oil exploration, earthquake forecasting, medical diagnoses, man-computer interaction), which couldn't be solved for decades and even centuries, were successfully resolved by using together computers and the Gestalt approach (Gelfand et al., 1989; Guberman et al., 1997; Rantsman and Glasko, 2004), but in order to use the principles, the rules, the notions, and the terms of Gestalt theory in computer applications, all of them have to be clearly defined.

The 100-year-long history of Gestalt theory development has not gone along the path projected by Wertheimer (1923). Wertheimer's followers neglected the spirit of his work, and preferred not the intensive way of developing Gestalt theory, but the extensive one—multiplying the number of basic principles and notions without appropriately defining them, spreading the vocabulary of Gestalt psychology to many other areas of science and engineering without valuable reasons.

The multiple failures in the development of Gestalt theory could have been avoided if Gestalt psychologists had paid more attention to Wertheimer's ideas, but this didn't happen. Many gestaltists use Wertheimer's name as a banner and a blessing from good science, and even put their own statements in his mouth.

Quote (from a most respectable review of the history of Gestalt psychology):

“In 1923, Wertheimer published a follow-up paper, which was an attempt to elucidate the fundamental principles of that organization. The most general principle was the so-called law of Prägnanz, stating, in its most general sense, that the perceptual field and objects within it will take on the simplest and most encompassing (‘ausgezeichnet’) structure permitted by the given conditions”. (Wagemans et al., 2012). The point is that in this paper Wertheimer didn't mention the “law of Prägnanz”, and didn't mention the word Prägnanz at all. It is not a surprise that Navon, van der Helm, Kimchi and some other prominent and active gestaltists in some of their papers on visual perception didn't mention Wertheimer's writing at all.

At the same time the deepest of Wertheimer's ideas were neglected, but they constitute the fundament of an alternative path of Gestalt psychology, which is really rooted in Wertheimer's thoughts and spirit.

**Figure 3:**
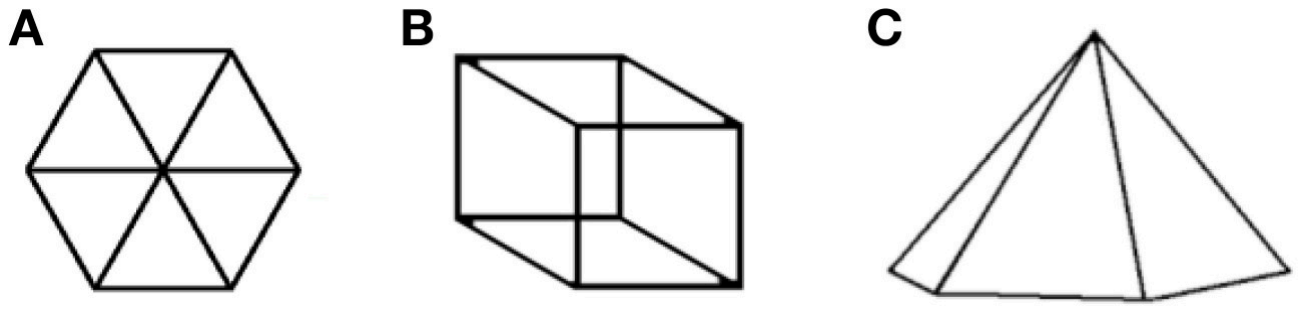
Three interpretations of stimulus **(A)** 1, flat figure; 2, Necker's cube, **(B)** 3, pyramid **(C)**.

1. Wertheimer understood Gestalt as a short description of the percept. Wertheimer introduced the term *Prägnanzstufen* when discussing the perception of all possible patterns of geometrical angles. He noted that some of the *Stufen*, which have a short description (acute, right, and obtuse), are privileged. The intermediate *Stufen* (angles) are difficult to describe in short words; they “are more indefinite in character”. Wertheimer used for the percepts of these *Stufen* the attribute Prägnanz, which means “a short description”, “in short words”. So, the Prägnanz is not the mystical tendency to the good Gestalt; it is the feature which allows us to recognize the good Gestalt. All experimental results in the Gestalt literature support this statement: all Gestalts are descriptions, and all good Gestalten are short descriptions. The most popular attribute of the good Gestalt—simplicity, which was never defined—can be substituted with the term short: the shortest description is always the simplest in a grammatical sense. The cause of the failure to define the meaning of “simple Gestalt” was that the Gestalt was treated as a visual object. This move, which resolves a number of problems, demands that **we switch from a visual domain to a linguistic one** (Guberman, 2015) It is worth to mention that van der Helm presented a similar idea: to choose the Gestalt, which has the simplest (i.e. the shortest) code (which is a description), (van der Helm, 2014) but, as I show in this paper, while we perceive the communicative stimuli we perceive only one percept (the good on) and the problem of choosing the best one from many percepts doesn't exist.
2. Wertheimer chose as a principal object of investigation the dotted and linear drawings not because of their simplicity, but because of their communicative nature—signals sent by one human being to another human being. As a matter of fact this position is a part of his broader view on Gestalt psychology. In his speech in 1924 on theoretical foundations of Gestalt psychology, Wertheimer stated: “When people are together as when they are at work, then the most unnatural behavior would be to behave as separate Egos. Under normal circumstances they work in common” (Wertheimer, 1924). This point of view was neglected by the mainstream of Gestalt psychology, but not completely lost: “Gestalt psychology is principally not referring to persons. It does not ask for subjects as causes of action. It rather tries to reveal dynamic relations applying to all persons involved” (Fitzek, 2013). From this point of view, by limiting the investigation of human perception to one person and isolating him from the whole (the society of humans), contemporary Gestalt Psychology contradicts the most fundamental idea of Gestalt Psychology: the importance of the whole in the interpretation of the part.
3. With awareness that the Gestalt theory was built on communicative stimuli (visual and audio), many problems of Gestalt theory can be resolved. A lot of attention was attracted by interpretation of ambiguous stimuli. One of them is presented in Figure 3A. If the question is: “which object of the physical world can be presented with this drawing”, there are three answers: (1) the hexagon with three diagonals, (2) the Necker cube, and (3) the hexagonal pyramid. But if the question is: “what figure did the author of this message intend to present”, there is only one answer: the hexagon with three diagonals. If the sender would like to show the cube, he would send the Figure 3B, and if he would like to show the hexagonal pyramid, he would send the Figure 3C.
  The communicative nature of the drawings dismissed the interpretation of these stimuli as a result of random generation proposed by some authors as a solid mathematical base of Gestalt psychology. Each stimulus appears not randomly, but it is created in a single example according to the unique design of the sender. The situation was explained by Laplace: “On a table we see letters arranged in this order, “Constantinople,” and we judge that this arrangement is not the result of chance, not because it is less possible than the others but because some person has thus arranged the aforesaid letters.”
4. Wertheimer understood the perception of communicative stimuli as an imitation of the act which produces the stimulus (the imitation principle).
  - The expression “good continuation” is applied to a line, so it is a “good continuation of the line” and is not applicable to the given image (because there is no intention to change it). The “good continuation” principle—one of the basic principles of Gestalt psychology—assumes that perception of a drawing includes the imaginable process of recreating (or imitating) the drawing (Guberman, 2007).
  - Wertheimer wrote: “In designing a pattern, for example, one has a feeling how successive parts should follow one another”. Here an imaginary action—“designing a pattern”—is used for explaining perception, and the process of redrawing the image is specified: creating genuine parts and drawing them in the right succession. Another quote—“Additions to an incomplete object (e.g., the segment of a curve) may **proceed** in a direction opposed to that of the original” is a clear description of redrawing the image.
  - When presenting dotted images, Wertheimer described them as lines (circles, arcs, zigzags) despite the fact that the stimulus contains no lines at all. As a matter of fact, it is a description of the way in which the image was created: while moving along an imaginable line, some dots were planted from time to time. The imitation principle is the realization of Wertheimer's goal—it overarched all other grouping principles (Guberman, 2015).
5. The imitation principle and the idea of good continuation inevitably emphasize the notion of the stroke—the line which was drawn without stops. The stroke is an elementary (minimal) block for creating linear drawings. For us our percept is the description of the process of redrawing the stimulus, it will be perceived in strokes. The rules of redrawing are simple:
  1. Each of two ends of a stroke is either free, or coincides with the end of another stroke. In the first case the redrawing proceeds to the beginning of the other stroke. In the second case the redrawing proceeds to the adjoining stroke.
  2. The crossing of two strokes is managed by the “good continuation” principle.

**Figure 4:**
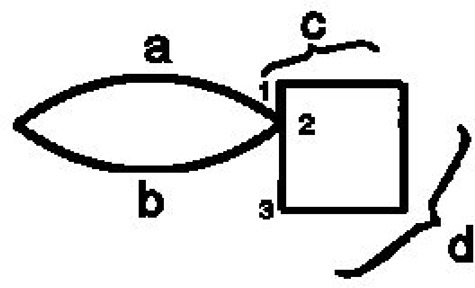
Following the strocks.

Accordingly, in the Figure 4 (from Wertheimer, 1923) we can start redrawing from the left-most point, and follow the arc a until we reach the point 2—the end of the stroke a. From the point 2 we can't follow to the point 3, because this point is not a beginning of a stroke, but is the middle point of the stroke 1–3. The only opportunity we have is to go along the stroke b and close the figure. It is important to note that we choose the arc b not because of the law of closure, but because following the imitation principle we have no other choice. Now we understand what Wertheimer meant when he wrote: “one has a feeling how successive parts should follow one another.”

**Figure 5:**
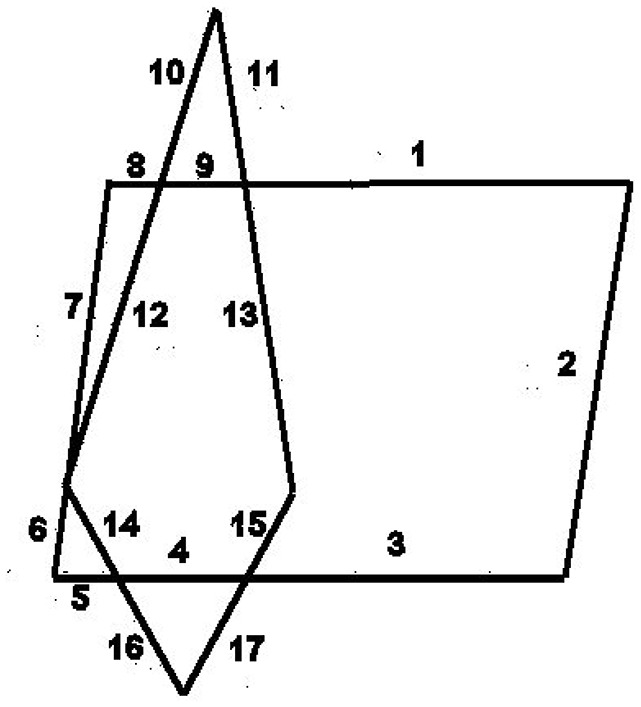
Following the strocks (Wertheimer, 1923).

**Figure 6:**
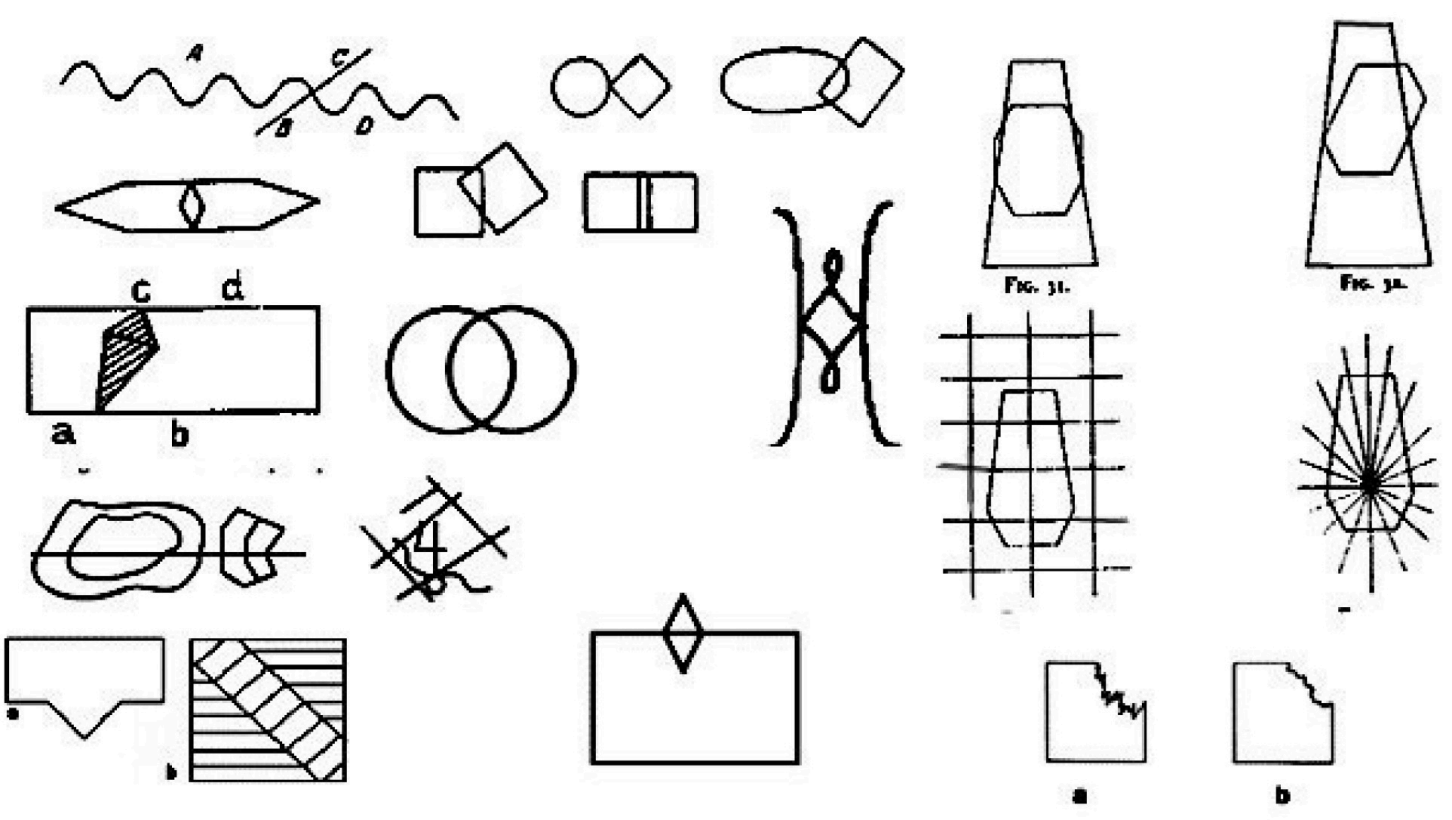
Collection of ambiguous figures.

In Figure 5 we perceive the parallelogram and the rhomboid, because that is what we get when following the strokes. If we start to redraw the Figure 5 from the right-upper corner of the parallelogram, we have to go to the end of the stroke - to the next corner, and continue following the other three sides of the parallelogram. We can see the same in Figure 6—the collection of “potentially ambiguous” linear drawings in Wertheimer's paper as well as in all other examples of “ambiguous” stimuli presented in the literature. Because all these drawings are perceived as sets of strokes, **we perceive only good Gestalts**. This is what Wertheimer meant: “one recognizes a resultant ‘good Gestalt’ simply by its own ‘inner necessity’” (Wertheimer, 1923).

It is remarkable that regular dictionaries explain the meaning of the stroke not only as a sign on paper (“a mark made by drawing a pen in one direction”), but as a pure movement as well: “an act of moving one's hand across a surface,” “a movement of the arms and legs in swimming,” “Move one's hand” (as a verb). It shows that instead of looking for mathematical decoration psychology has to look in the opposite direction—to the theories of liberal arts (linguistics in particular), where very smart people are investigating human perception of the world. In the 1920s in Berlin, there emerged the school of structural linguistics. It is not a surprise that Gestalt psychology left its mark on this movement (which later gave birth to Russian formalism and the Prague school). The co-founder of structural linguistics (together with R. Jacobson) B. Shklovsky wrote: “The form is the rule of object's construction,” i.e., the sense of the form is how it was done.

Consequently, we don't perceive bad Gestalts—the ugly prolix descriptions. They are not products of our perception, they are products of an *intellectual act* (in Mach's words), cutting the drawing into pieces. Our perception doesn't search in the sea of bad Gestalten for the best one, and it is not driven by the mystical force of *Prägnanz* to the Holy Grail of Gestalt theory—to the good Gestalt.

The imitation principle and the underlying neuronal mechanism establish a connection between our psychology and our physical body. Wertheimer foresaw it. He also understood that the body-mind relations were always a part of the philosophical fight between materialism and idealism. Discussing the essence of Gestalt, Wertheimer wrote: “In the opinion of many people the distinction between idealism and materialism implies that between the noble and the ignoble. What is there so repugnant about the materialistic and mechanical? What is so attractive about the idealistic? In terms of specific problems one soon realizes how many bodily activities there are which give no hint of a separation between body and mind. When a man is timid, afraid or energetic, happy or sad, it can often be shown that the course of his physical processes is Gestalt-identical with the course pursued by the mental processes” (Wertheimer, 1924). The last states that the Gestalten of the physical movements of our body are identical to Gestalten of our perception. The imitation theory explains that the final percept—the Gestalt—belongs not to the visual domain, but to the motor domain, i.e. the Gestalt is **not identical** to the physical movement—**it is** a physical movement.

Wertheimer's objection to the idealistic interpretation of perception is an argument against the philosophy of holism, which claims dominance of the whole, global precedence, configural superiority, “forest before trees” and other unsuccessful attempts to soar over the science.

This is how Gestalt theory looks from Wertheimer's perspective – simple and definite.

## Gestalt theory in wertheimer's perspective

**Whole**: the general meaning – “something that consists of parts”.

In Gestalt theory – an object of perception that consists of parts.

**Part**: the general meaning – “piece that combines with other pieces to form the whole”.

In Gestalt theory – *set of pieces, which provides the shortest description of the whole* (the good Gestalt), is **the set of parts** that constitute the given whole.

The center of gravity of the theory of Gestalt perception is shifted from **searching for the good Gestalt** to **finding the adequate partition**.

How does our brain find the adequate parts when receiving the communicative stimulus?

- For linear drawings we transfer the input signal from visual domain into motor domain. As a result we perceive the input as a sequence of elementary movements - strokes. Here the perceived complex of motor command was matched to the library of motor commands accumulated during the past drawing (or writing) activity. Some complexes of movements of such activity have names, from which the final linguistic answer is constructed. Another part of the answer comes from visual channel and defined the spatial relations between recognized wholes (“two intersecting circles”). Now the response to the question “What do you see?” is ready. Following the rules of tracking the strokes (described above) we always perceive the percept in adequate parts (as Kohler called it – “natural” parts). Examples are presented in Figure 6.
- For handwriting we also transfer the static image into a sequence of elementary movements (six in total): 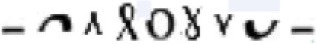. As geometrical figures these elements belong to different classes: with the hole and without, with the cross and without, consists of one or two strokes etc. But as movements the neighbors in the sequence are very similar (for instance 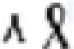 and 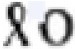). Accordingly, different variations of a (
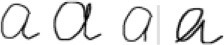
) are perceived as very similar sets of motor commands, while their geometrical descriptions are quite different. This is why 30 years of attempts to create the computer program for cursive handwriting recognition using the geometrical approach were unsuccessful. The first really working program to appear on the market was based on the imitation principle and operated in the motor domain (Guberman, 1995), and was licensed by main computer companies (Apple Co, Microsoft, Siemens).
- We perceive speech in the motor domain as a complex of motor commands to the muscles of the articulatory tract (imitating the sender's activity).

In general, the consonants and the vowels are produced by different sets of muscles. For example, upper and lower labial tractors, which make the lips flattened and stretched, are involved in creating the vowel (i), and never participate in creation of any vowel. The muscles which move the lips in the vertical direction participate in creation of labial consonants only. The independence of creating the vowels and the consonants allows us to generate speech not as sequence of consonants and vowels, but in parallel: each consonant appears in the background of a vowel (Andreevsky and Guberman, 1996). Despite the fact that the motor commands for vowels and consonants are independent, the resulting geometry of the articulation tract (which defined the audio signal) at any moment in time is influenced by both sounds (the consonant and the vowel). This phenomenon is called coarticulation, and constitutes the main difficulty in computer speech recognition. Due to the mechanism of mirror neurons, out brain perceives speech as a sequence of motor commands: separately for the group of muscles executing the articulation of consonants and the different group of muscles executing the vowels. This allows us to resolve two fundamental problems of linguistics: (1) the interaction between vowels and consonant, to which Buhler referred as “phenomenon of the syllable,” and called it “the central point to the make-up of speech”(Bühler, 1990), and (2) the definition of the notion of phoneme as an act of innervation of the particular group of muscles. The latter is in the opposition to the claims of the Prague school of structural linguistics, which claims that the notion of phoneme belongs to audio domain: “Phoneme is a class of sounds” (S. Trubetskoy), “Phoneme is the psychical equivalent of the sound” (Baudouin de Courtenay) etc., but can't agree on its definition.

The ability of the imitation principle to resolve cardinal problems in perception in the visual as well as in the audio domain demonstrates how deeply it is rooted in the processes of perception.

## Conclusion

From the very beginning and in the following 100 years Gestalt psychology influenced the whole of psychology and many humanitarian sciences as well. At the very beginning Wertheimer himself pointed out the weaknesses of the newborn Gestalt theory: it had multiple basic principles and weak definitions. Despite these problems being forever criticized, they have not been solved—until now.

Analysis of Wertheimer's works allows us to reconstruct the Gestalt theory (at least the part concerning perception of communicative signals-drawings, speech and music), and overcome the weaknesses of Gestalt Psychology: define the main notions, introduce the general principle of perception which overarches all known laws and unifies different Gestalt phenomena (handwriting, drawings, speech, music, and apparent movement)—**the imitation principle**. The presented model of perception is supported by fundamental neurophysiological data—the mirror neurons phenomenon and simulation theory.

This is how Gestalt theory looks from Wertheimer's perspective – simple and definite.

1. The whole is an object of perception, which consists of parts.
2. The whole can be presented as divided in pieces in various ways. The set of pieces, which provides the shortest description of the whole (the good Gestalt), is the set of parts that constitute the given whole.
3. The interpretation of the whole and the interpretation of parts have to be mutually concerted.
4. Perception is a process of reconciliation of the interpretations of the whole and of the parts.
5. The minimal part of the percept of a linear drawing is a stroke.
6. The Gestalt is the description of the sequence of actions which we will use to redraw the stimulus – the imitation principle.
7. The imitation principle unites vision, speech, music, and apparent motion as Gestalt phenomena.

## Author contributions

The author confirms being the sole contributor of this work and approved it for publication.

### Conflict of interest statement

The authors declare that the research was conducted in the absence of any commercial or financial relationships that could be construed as a potential conflict of interest.

## Footnotes

^1^Gestalt psychology has many connections with structural linguistics for both pay particular attention to the notion of structure. More then 100 years ago de Saussure (who inspired the emergence of structural linguistics) in his theory of sign emphasized the danger of confusing the “signified” with “referent”: The former is a “mental concept”, the latter the “actual object” in the (world). Similar confuse repeatedly appeared in Gestalt literature (de Saussure et al., 1966).
